# Full Activation of Kinase Protein Kinase B by Phosphoinositide-Dependent Protein Kinase-1 and Mammalian Target of Rapamycin Complex 2 Is Required for Early Natural Killer Cell Development and Survival

**DOI:** 10.3389/fimmu.2020.617404

**Published:** 2021-02-09

**Authors:** Junming He, Jun Zhao, Yuhe Quan, Xinlei Hou, Meixiang Yang, Zhongjun Dong

**Affiliations:** ^1^ School of Medicine and Institute for Immunology, Tsinghua University, Beijing Key Lab for Immunological Research on Chronic Diseases, Tsinghua University, Beijing, China; ^2^ Zhuhai Precision Medical Center, Zhuhai People’s Hospital (Zhuhai Hospital Affiliated with Jinan University), Jinan University, Zhuhai, Guangdong, China; ^3^ The Biomedical Translational Research Institute, Faculty of Medical Science, Jinan University, Guangzhou, Guangdong, China

**Keywords:** natural killer (NK) cell, protein kinase B (PKB), phosphoinositide-dependent protein kinase-1 (PDK1), mammalian target of rapamycin complex 2, development, survival

## Abstract

The role of PI3K-mTOR pathway in regulating NK cell development has been widely reported. However, it remains unclear whether NK cell development depends on the protein kinase B (PKB), which links PI3K and mTOR, perhaps due to the potential redundancy of PKB. PKB has two phosphorylation sites, threonine 308 (T308) and serine 473 (S473), which can be phosphorylated by phosphoinositide-dependent protein kinase-1 (PDK1) and mTORC2, respectively. In this study, we established a mouse model in which PKB was inactivated through the deletion of PDK1 and Rictor, a key component of mTORC2, respectively. We found that the single deletion of PDK1 or Rictor could lead to a significant defect in NK cell development, while combined deletion of PDK1 and Rictor severely hindered NK cell development at the early stage. Notably, ectopic expression of myristoylated PKB significantly rescued this defect. In terms of mechanism, in PDK1/Rictor-deficient NK cells, E4BP4, a transcription factor for NK cell development, was less expressed, and the exogenous supply of E4BP4 could alleviate the developmental defect of NK cell in these mice. Besides, overexpression of Bcl-2 also helped the survival of PDK1/Rictor-deficient NK cells, suggesting an anti-apoptotic role of PKB in NK cells. In summary, complete phosphorylation of PKB at T308 and S473 by PDK1 and mTORC2 is necessary for optimal NK cell development, and PKB regulates NK cell development by promoting E4BP4 expression and preventing cell apoptosis.

## Introduction

Natural killer (NK) cells are important innate lymphocytes capable of mediating both cytotoxicity and cytokine production in response to tumor cells or virus-infected cells, as well as rejecting major histocompatibility complex class I (MHC-I)-mismatched allogeneic bone marrow (1, 2). NK cells are derived from bone marrow hematopoietic stem cells (HSCs) through a gradual process, including common lymphoid progenitor (CLP), NK progenitor (NKp), immature NK (iNK), and mature NK cells (mNK). NK cell development and homeostasis strictly rely on pleiotropic cytokine IL-15 and its signaling. Mice lacking either IL-15 or any of the IL-15 receptor subunits almost have no detectable NK cells (3, 4). IL-15 mainly activates phosphatidylinositol-4, 5-bisphosphate 3-kinase (PI3K) and mammalian target of rapamycin (mTOR) pathway (5). Mice simultaneously lacking the PI3K subunits P110*γ* and δ exhibit a severe impairment in early NK cells development and function (6, 7).We previously demonstrated that phosphoinositide-dependent kinase 1 (PDK1), a kinase connecting PI3K and mTOR, is essential for NK cell development by inducing transcription factor E4BP4 and maintaining IL-15 responsiveness (8). Ablation of mTOR affects NK cell blastogenesis, activation, and effector functions (9). mTOR binds to Raptor and Rictor to form two complexes, mTORC1 and mTORC2. mTORC1 has been shown to play an active role in the early and later stages of NK cell development, promoting the development and function of NK cells, and it can also regulate mTORC2 activity by maintaining the IL-15 signaling (9, 10). Using gene-targeting technique based on Ncr1-Cre mice, recent studies have shown the deletion of mTORC2 at the terminal stage of NK cells did not affect the transcriptional regulation of NK cells, but it can inhibit the function of NK cells by inhibiting mTORC1 (9, 11).

As a central regulator, the serine/threonine kinase PKB/Akt links the upstream PI3K with the downstream mTOR signaling, and converts the environmental signals into cellular response signals. To date, three PKB family members have been identified in mammals, designated PKB1, PKB2 and PKB3, which share similar domain structure and function redundantly. The germline deletion of PKB leads lethal disorder. The contributions of PKB to immune cells such as T cells (12), B cell (13) and macrophages (14) had been reported. However, there is no clear genetic study to address the global role of PKB in NK cell development, perhaps due to the potential redundancy of PKB.

The activity of PKB is delicately modulated by the phosphorylation of two sites, Thr308 and Ser473. The first step for PKB activation is the phosphorylation of Thr308 by PDK1. This process is mediated by the tethering of PKB and PDK1 to the plasma membrane (15). The lipid second messenger phosphatidylinositol 3,4,5-trisphosphate (PIP3), produced by the class I PI3Ks, binds directly to the pleckstrin homology (PH) domain of PKB, driving a conformational change in the molecule, which enables the activation loop of PKB at Thr308 to be phosphorylated by PDK1 (16). Furthermore, the full activation of PKB probably need the phosphorylation of Ser473 by mTOR complex 2 (mTORC2) (17). Limited studies demonstrated that PDK1 and Rictor play a synergistic role in PKB activation (18). However, it remains unknown whether these two molecules are synergistically required for immune cells, such as NK cell development.

To investigate the role of PKB in early NK cell development, we used newly generated *CD122^Cre/+^* mice to established a mouse model in which PKB was inactivated through the deletion of PDK1 and Rictor, a key component of mTORC2, respectively. In *CD122^Cre/+^* mice, Cre begins to be expressed during and after NK cell commitment. We demonstrated that complete phosphorylation of PKB at T308 and S473 by PDK1 and mTORC2 is necessary for optimal NK cell development. Mechanistically, PKB regulates NK cell development by promoting E4BP4 expression and preventing cell apoptosis.

## Materials and Methods

### Mice

*PDK1^flox/flox^* mice were a gift from Dario Alessi (University of Dundee). CD122-Cre mice was described previously (19). *Rictor^flox/flox^* mice, β2m-deficient mice were purchased from Jackson Laboratory. The lines of conditional PDK1, or Rictor knockout mice, in which the *PDK1* or *Rictor* gene was deleted at the NKp stage, were generated by crossing *PDK1^fl/fl^* mice or *Rictor^fl/fl^* mice with mice carried CD122-Cre. *PDK1* and *Rictor* double deficient mice, in which the *PDK1* and *Rictor* genes were deleted at the NKp stage, were generated by crossing *PDK1^fl/fl^CD122^Cre/+^* mice with *Rictor^fl/fl^* mice. *PDK1^fl/fl^*, *Rictor^fl/fl^*, and *PDK1^fl/fl^Rictor^fl/fl^* mice were used as control. Sex- and age-matched female and male mice (8-12 weeks) were used in our experiment. All mice were bred and maintained in specific pathogen-free animal facilities of Tsinghua University. All animal procedures were approved by the Animal Ethics Committee of Tsinghua University.

### Flow Cytometry

Monoclonal antibodies against mouse CD3 (17A2), NKp46 (29A1.4), NK1.1 (PK136), CD127 (SB/199), Ly49A (A1), Ly49H (3D10), Ly49G2 (4D11), Ly49D (4E5), NKG2A (20D5), NKG2D (CX5), KLRG1 (2F1), CD11b (M1/70), CD27 (LG.7F9), Bcl-2 (10C4), E4BP4 (S2M-E19), Eomes (Dan11mag), T-bet (4B10), Ki67 (B56) and isotype controls were purchased from eBioscience. Anti-Ly49C/I (YLI-90) and anti-phospho-STAT5 (pY694) were purchased from BD Biosciences. Anti-phospho-S6 (D57.2.2E). Anti-phospho-AKT T_308_ (D25E6) and anti-phospho-AKT S_473_ (D9E) were obtained from Cell Signaling Technology. For the detection of phosphorylated signaling proteins, NK cell was fixed with Phosflow Lyse/Fix buffer, permeabilized with Phosflow Perm buffer III (eBioscience), and then stained with antibodies. Data were acquired on an LSRII (Becton Dickinson) flow cytometer and analyzed using FlowJo software (Treestar). The expression level was presented as percentage or net MFI, which was determined by subtracting the mean fluorescence intensity of isotype control.

### *In Vitro* Detection of Natural Killer Cell Responsiveness to IL-15

To detect NK cell responsiveness to IL-15, splenocytes were incubated in 24-well plates (2×10^6^ cells in 500 µL 1640 medium) with the stimulation of rmIL-15/IL-15Rα complex (10 ng/ml). Twenty-four hours later, cells were collected for flow cytometry analysis. Meanwhile, spleen lymphocytes without stimulation were treated with the same staining, then used as control.

### Western Blotting

NK cells were purified from splenocytes using a positive selection kit (StemCell Technologies) and then cultured in RPMI 1640 medium containing 20% fetal bovine serum, β-Mercapto alcohol, and 1,000 U/ml IL-2. Seven days later, NK cells were further stimulated with or without rmIL-15/IL-15Rα complex (10 ng/ml) for 24 h. Cells were collected and the protein was extracted as previously described. Equal amounts of protein were fractionated by 12% SDS-PAGE and transferred to polyvinylidene difluoride membrane. Membranes were blocked with TBS/Tween 20 (TBST) containing 5% bovine serum albumin (BSA) for 1 h. After blocking, the membranes were incubated with the appropriate specific primary antibodies at 4°C overnight, followed by incubation with HRP-conjugated secondary antibodies (Beyotime, Shanghai). Detection was performed using an enhanced chemiluminescence kit (Thermo Scientific, Hudson, NH, USA) according to the manufacturer’s instructions. Densitometry analysis was performed using ImageJ software.

### Detection of Natural Killer Cell Apoptosis

NK cell Caspase activity was measured with fluorescein isothiocyanate (FITC)- conjugated z-VAD-fmk according to the manufacturer’s instruction (eBioscience).

### Bone Marrow Reconstitution

Mice were treated with 5-FU for 4 days, and bone marrow cells were collected for spin-infection with MSCV retrovirus encoding target genes. The infected BM cells were then transferred into recipient mice that were sub-lethally irradiated. After eight weeks, the reconstruction of recipients was assessed by flow cytometry.

### *In Vivo* Splenocytes Rejection Assay

Splenocytes from *β2m*-deficient mice were depleted of red blood cells by Ficoll-Hypaque density gradient centrifugation and then labeled with 5µM CFSE (Molecular Probes). At the same time, splenocytes from C57BL/6 mice were labeled with 0.5µM CFSE (10-fold less than *β2m* cells). Two types of CFSE-labeled splenocytes were mixed at a 1:1 ratio. A mixture of 2×10^6^ splenocytes was intravenously injected into mice pre-treated with 200 µg Poly (I:C). Eighteen hours later, CFSE-positive cells in the spleen and lymph nodes were determined by flow cytometry.

### *In Vivo* RMA-S Clearance Assay

Mice treated with 200 µg Poly (I:C) for 18 h were intraperitoneally injected with a mixture of tumor cells, NK-sensitive cells RMA-S transfected with GFP (10^6^), and NK-non-sensitive cells RMA expressing DsRed (10^6^). Tumor cells were mixed at a 1:1 ratio. Eighteen hours later, the mice were sacrificed, and cells in the peritoneal cavity were collected by repeated washing with PBS containing 2 µM EDTA. The relative percentage of residual RMA-S-GFP and RMA-DsRed cells were measured by flow cytometry.

### B16 Melanoma Lung Metastasis Mouse Model

B16F10 melanoma cells in the log phase were resuspended in 1×PBS and intravenously injected into the mice (2×10^5^ cells/mouse). Fourteen days later, the mice were sacrificed. The lung was weighed and the number of lung surface nodules was counted under a dissecting microscope.

### Statistical Analyses

Statistical significance was determined using Prism software. Two-tailed unpaired or paired Student’s t-tests between two groups and two-way analysis of variance (ANOVA) across multiple groups were used to determine significance. A *p* value of less than 0.05 was considered significant. *p<0.05, **p<0.01, ***p<0.001, ****p<0.0001.

## Results

### Deletion of Phosphoinositide-Dependent Protein Kinase-1 Abolishes Protein Kinase B T308 Phosphorylation But Enhances Protein Kinase B S473 Phosphorylation in Natural Killer Cells

**Figure 1:**
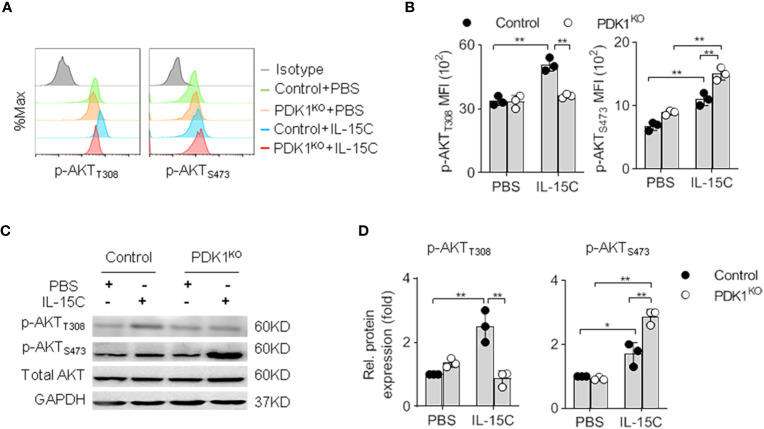
Deletion of PDK1 abolishes PKB T308 phosphorylation but enhances PKB S473 phosphorylation in NK cells. **(A, B)** The expression levels of AKT phosphorylated at T308 (p-AKT_T308_) and S473 (p-AKT_S473_) in Control or PDK1-deficient NK cells were determined by flow cytometry after IL-15C stimulation. Representative overlaid histograms **(A)**, absolute MFI for p-AKT_T308_ and p-AKT_S473_ **(B)**. **(C, D)** The expression levels of p-AKT_T308_ and p-AKT_S473_ in Control or PDK1-deficient NK cell were analyzed by Western blotting **(C)** and densitometry **(D)** after IL-15C stimulation. *p < 0.05, **p < 0.01.

We previously showed that PDK1 deletion at the NK progenitor (NKp) stage caused a reduction of NK cell number in PDK1*^fl/fl^/*CD122^Cre/+^ (PDK1^KO^) mice. However, there were still residual NK cells in the spleen and bone marrow (19). To investigate the reason for residual NK cell survival, we examined the alteration of PI3K-mTOR signaling in these cells. We chose the phosphorylation of PKB as a readout of IL-15 signaling activation. As previously reported, the phosphorylation of PKB T308 and PKB S473 were significantly increased following IL-15/IL-15Rα complex (IL-15C) stimulation in Control NK cells. However, IL-15C could not boost the phosphorylation of PKB at T308 in PDK1-deficient NK cells (**Figures 1A, B**). Thus, the survival of remaining NK cells may not be due to the inefficiency of PDK1 deletion in NK cells.

Our group had reported that the deletion of PDK1 on NK progenitor diminished mTORC1 activation in NK cells (19). Yang et al. revealed that the deletion of PDK1 in the heart resulted in a dramatic enhancement of PKB S473 phosphorylation (18). These studies prompted us to test whether blockage of PDK1-mTORC1 signaling affected the phosphorylation level of PKB S473 in NK cells. Intriguingly, flow cytometry assay revealed that the amount of PKB S473 phosphorylation was increased upon PDK1 deletion in NK cells (**Figures 1A, B**). To confirm this experiment, we purified NK cells from control or PDK1-deficient mice to run western blot assay. Accordingly, a similar conclusion was obtained (**Figures 1C, D** **)**. Therefore, combined with the published data, we suggest that the inactivation of PDK1 leads to the increased activation of PKB S473, which may be a compensating effect.

### Deletion of Rictor Abolishes the Enhanced Phosphorylation of Protein Kinase B S473 in Phosphoinositide-Dependent Protein Kinase-1-Deficient Natural Killer Cells

**Figure 2:**
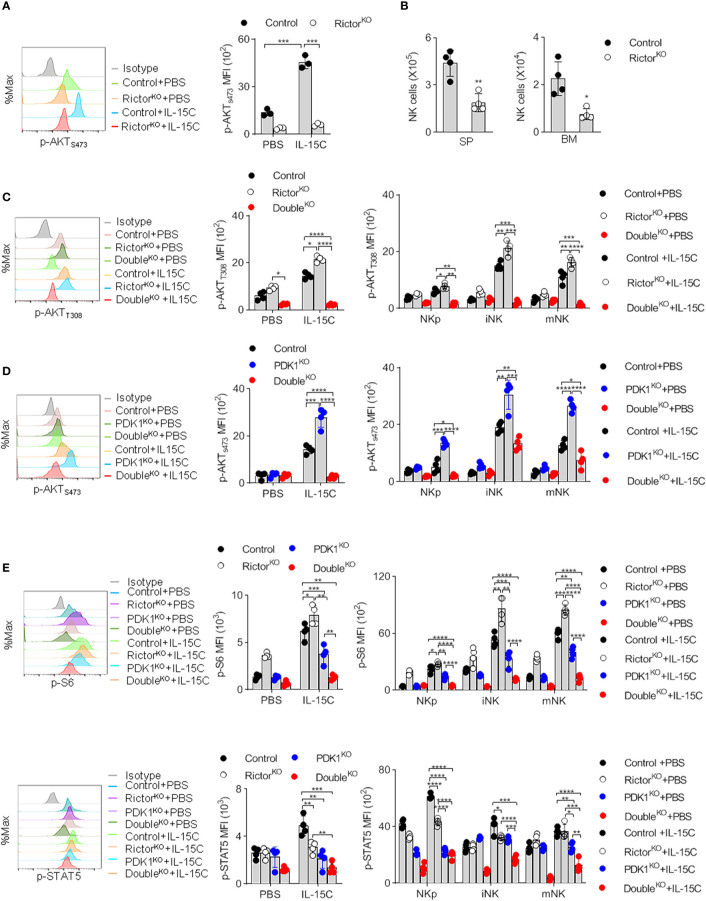
Deletion of Rictor abolishes the enhanced phosphorylation of PKB S473 in PDK1-deficient NK cells. **(A)** The expression levels of p-AKT_S473_ in Rictor-deficient NK cell were detected by flow cytometry after IL-15C stimulation. **(B)** The number of NK cell in the spleen (SP) and bone marrow (BM) of Rictor-deficient mice was counted. **(C)** The expression levels of p-AKT_T308_ in Control, Rictor-deficient or PDK1/Rictor double deficient NK cells were detected by flow cytometry after IL-15C stimulation. **(D)** The expression levels of p-AKT_S473_ in Control, PDK1-deficient or PDK1/Rictor double deficient NK cells were determined by flow cytometry after IL-15C stimulation. **(E)** The expression levels of p-S6 and p-STAT5 in spleen NK cell were detected by flow cytometry after IL-15C stimulation in indicated mice. Representative overlaid histograms (left), absolute MFI (right).The data represent one of three independent experiments, n≥3 for each experiment, and values are expressed as the mean ± SD. *p < 0.05, **p < 0.01, ***p < 0.001, ****p < 0.0001.

Recently, Wang et al. reported that Rictor could promotes NK cell specification and maturation (10). To consolidate this notion in our system, we generated *Rictor^fl/fl^/CD122^Cre/+^* mice (Rictor^KO^) to disrupt Rictor in NKp cells. Rictor deletion nearly abolished PKB S473 phosphorylation (**Figure 2A**). Consistent with the published data, we found that Rictor-deleted mice exhibited a decreased NK cells number (**Figure 2B**). We also found that phosphorylation of T308 of PKB increased in the NK cells isolated from Rictor^KO^ mice (**Figure 2C**). Thus, we further confirmed the role of mTORC2 in NK cell development using CD122-Cre that Rictor was deleted from NKp stage.

To reveal the reason for the enhanced phosphorylation of PKB S473 in PDK1-deficient mice, we generated *PDK1^fl/fl^/Rictor^fl/fl^/CD122^Cre/+^* mice (Double^KO^). As expected, the deletion of Rictor in PDK1^KO^ mice could notably diminish the enhanced phosphorylation level of PKB S473 in total and NK cell subsets (**Figure 2D**). The PDK1 and Rictor double deficiency abolished the phosphorylation of PKB T308 and PKB S473 in NK cells (**Figures 2C, D** **)**. Interestingly, the additional deletion of Rictor also decreased the amount of phosphorylated form of S6 and STAT5 in total and different NK cell subsets (**Figure 2E**), suggesting a cooperative role of PDK1 and Rictor in IL-15-induced signaling. These results demonstrated that the increased PKB S473 phosphorylation in PDK1-depleted NK cells was mediated by mTORC2.

### Mammalian Target of Rapamycin Complex 2 Deficiency Aggravates the Developmental Defect of Phosphoinositide-Dependent Protein Kinase-1-Deficient Natural Killer Cells

**Figure 3:**
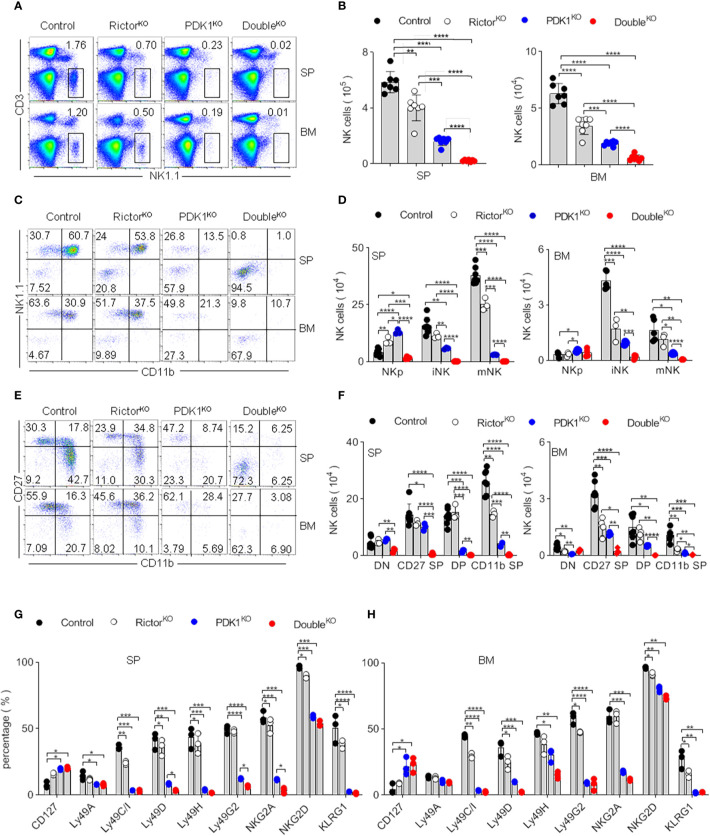
mTORC2 deficiency aggravates the developmental defect of PDK1-deficient NK cells. **(A, B)** Representative flow cytometric plots **(A)** and the absolute number **(B)** of NK cell (CD3^-^NK1.1^+^) in the SP and BM of the indicated mice. **(C, D)** Representative flow cytometric plots **(C)** and the number **(D)** of NK cell subsets in the SP and BM of annotated mice. NKp (CD122^+^NK1.1^-^CD11b^-^), iNK (CD122^+^NK1.1^+^CD11b^-^), mNK (CD122^+^NK1.1^+^CD11b^+^). **(E, F)** Representative flow cytometric plots of CD27 and CD11b expression **(E)** and number of stage-specific NK cell **(F)** in the SP and BM of annotated mice. DN (CD27^-^CD11b^-^), CD27 SP (CD27^+^CD11b^-^), DP (CD27^+^CD11b^+^) and CD11b SP (CD27^-^CD11b^+^). **(G, H)** Statistical results of percentage in NK cell membrane receptor expression spectra in SP **(G)** and BM **(H)** of indicated mice. The data represent one of three independent experiments, n≥3 for each experiment, and values are expressed as the mean ± SD. *p < 0.05, **p < 0.01, ***p < 0.001, ****p < 0.0001.

To investigate whether mTORC2 activity contributes to the survival of remaining NK cells in PDK1-deficient mice, we monitored the frequency and number of NK cells in mice lacking PDK1/Rictor. As shown in **Figures 3A, B**, the individual deletion of Rictor or PDK1 reduced the proportion and number of NK cells in the spleen and bone marrow. Compared with the absence of Rictor, the loss of PDK1 might have a more significant impact on the development of NK cells, indicating that the development of NK cells is more dependent on PDK1. However, the simultaneous deletion of these two molecules almost made NK cells undetectable in the spleen and bone marrow. Therefore, these data suggest that mTORC2 plays an important role to keep the survival of NK cells in PDK1-deficient mice.

Differentiation of NK cell progenitors into mature NK cells sequentially takes several steps. We previously reported that the early loss of PDK1 mainly affects the development of NK cells (19). To investigate whether Rictor deficiency aggravated the development of NK cells with PDK1 deficiency at the same temporal and spatial levels, we quantified the relative proportion of NK cell subsets. According to NK1.1 and CD11b expression, NK cells were divided into three developmental stages: CD122^+^NK1.1^–^CD11b^–^ NK progenitor (NKp), CD122^+^NK1.1^+^CD11b^–^ immature NK (iNK) and CD122^+^NK1.1^+^CD11b^+^ mature NK (mNK) (**Figure S1A**). The PDK1 or Rictor deficiency did not affect the expression of CD122 in NKp cells (**Figures S1B, C**). Notably, the tiny trackable population of NK cells retained in PDK1 and Rictor DKO mice almost was NKp. iNK and mNK cells were almost not detectable in these mice (**Figures 3C, D**). It had confirmed that the maturation of NK cell is a 4-stage process, according to the expression level of CD27 and CD11b (20, 21). We also evaluated this process through the expression level of CD27 and CD11b in our study. In PDK1-deficient mice, most of NK cell was arrested at CD11b^–^CD27^–^ DN and CD27^+^ SP stages. In contrast, almost all the traceable cells in DKO mice were at CD11b^–^CD27^–^ DN stages (**Figures 3E, F**). These analyses suggest that mTORC2 and PDK1 synergistically regulate NK cell development during the early stage, most likely at the NKp commitment stage.

To prove the above point, we examined the repertoire of NK cell receptors, which is sequentially acquired by NK cells during their development. CD127 was usually expressed only on a fraction of early NK cells in Control mice. However, the proportion of CD127 positive cells was increased in PDK1 and Rictor DKO mice. Ly49 family members (including Ly49A, C/I, D, H, G2) and KLRG1, which represent NK cell mature and senescence respectively, were hardly expressed in PDK1 and Rictor DKO NK cells (**Figures 3G, H**). These data further support the notion that mTORC2 and PDK1 likely co-regulate the early development of NK cells.

### The Loss of Mature Natural Killer Cells in Phosphoinositide-Dependent Protein Kinase-1/Rictor-Deficient Mice Leads to the Impairment of Natural Killer Cell Surveillance *In Vivo*

**Figure 4:**
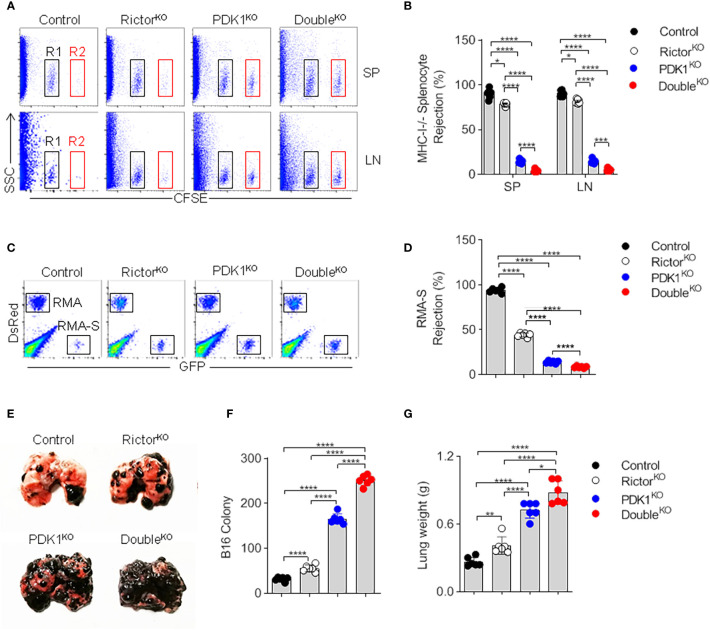
NK cells fail to mediate *in vivo* surveillance in PDK1/Rictor-deficient mice. **(A)** Representative flow cytometry plot of CFSE^+^ cells obtained from the SP and Lymph nodes (LN) of the indicated recipient mice at 18 h after injection with an equal number of C57BL/6 and *β2m*-deficient splenocytes labeled with various concentrations of the cytosolic dye CFSE. R1, CFSE-low splenocytes from C57BL/6 mice; and R2, CFSE-high splenocytes from *β2m*-deficient mice. **(B)** The percentages of rejected *β2m*-deficient splenocytes from the SP and LN of the indicated recipient mice. **(C)** Representative flow cytometry plot of injected RMA-S cells in the peritoneal cavity at 18 h after intraperitoneal injection of the indicated mice with a mixture of NK cell-sensitive RMA-S cells expressing green fluorescent protein (GFP) together with NK cell-resistant RMA cells expressing the fluorescent protein DsRed. **(D)** The percentage of rejected RMA-S cells. **(E–G)** B16 metastasis assay, the indicated mice were injected intravenously with 2**×**10^5^ B16 cells. The mice were sacrificed 14 day later, and the lung weights and numbers of tumor nodules were counted. Each symbol represents an individual mouse. Data are shown as means ± SD and represent one of three independent experiments, n≥3 for each experiment. *p < 0.05, **p < 0.01, ***p < 0.001, ****p < 0.0001.

To confirm the impaired development and reduced number of NK cells in PDK1- or/and Rictor-deficient mice, we assessed the ability of NK cells to mediate “missing-self” rejection. The results showed that MHC-I-missing splenocytes were largely eliminated by NK cells in Control mice. However, NK cell-mediated killing was significantly reduced in PDK1- or Rictor-deficient mice and was more significant in PDK1- and Rictor-deficient mice (**Figures 4A, B**). We also examined NK cell-mediated rejection of RMA-S cells, an NK-sensitive tumor cell line. Compared with Control mice, PDK1 and Rictor double deficient NK cells showed serious defects in RMA-S clearance (**Figures 4C, D**). Finally, the ability of NK cell to suppress the metastasis of B16 melanoma exhibited severer in the PDK1 and Rictor double-deficient mice than that in Rictor^KO^ or PDK1^KO^ mice (**Figures 4E–G**). Thus, we conclude that in PDK1- and Rictor-deficient mice, the absence of functional NK cells dramatically impairs NK cell-mediated rejection of “unwanted” cells.

### The Ectopic Expression of Myristoylated Protein Kinase B Corrects the Defect of Natural Killer Cell Development

**Figure 5:**
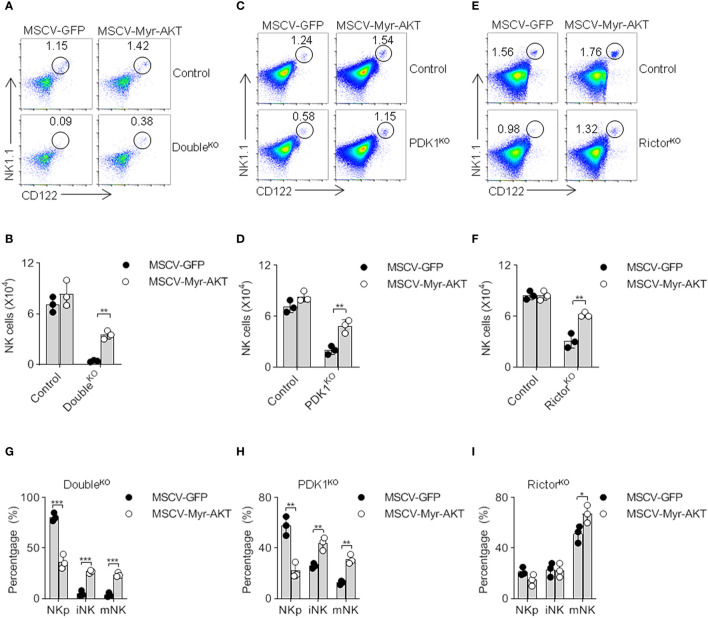
The ectopic expression of myristoylated PKB corrects the defect of NK cell development. **(A–F)** Double- **(A, B)**, PDK1- **(C, D)** or Rictor-deficient **(E, F)** mice were treated with 5-FU for 4 days, and bone marrow cells were collected for spin-infection with MSCV retrovirus encoding control GFP, or Myr-AKT (activated-PKB). The infected BM cells were then transferred into recipient mice. After 8 weeks, CD122 vs NK1.1 expression on gated CD3^−^ cells was analyzed by flow cytometry. The numbers in the outlined areas indicate the percent of CD3^−^CD122^+^NK1.1^+^ cells **(A, C, E)**. Numbers of CD3^−^CD122^+^NK1.1^+^ cell are enumerated **(B, D, F)**. **(G–I)** The developmental stages of NK cell was analysed after replenish Myr-AKT to HSC of Double-, PDK1- or Rictor-deficient mice **(G–I)**. The data represent one of three independent experiments, n≥3 for each experiment, and values are expressed as the mean ± SD. *p < 0.05, **p < 0.01, ***p < 0.001.

Considering that PKB activity is largely determined by phosphorylation mediated by PDK1 and mTORC2, we next verified whether the reduction in PKB activation resulted in the severe developmental defect of PDK1/Rictor-deficient NK cells. Previous studies have demonstrated that the myristoylated PKB, also known as Myr-PKB, promotes PKB to become membrane-anchored, while PKB carrying mutations in T308D and S473D may reduce its dependence on phosphorylation mediated by two upstream kinases, PDK1 and mTORC2 (22). Thus, we produced recombinant retroviruses expressing Myr-PKB mutants, here we named it as activated-PKB (Myr-AKT in **Figure 5**). Through bone marrow reconstitution experiments, we revealed that the exogenous expression of active PKB significantly increased the proportion and the absolute number of NK cells (**Figures 5A–F**). Through an in-depth analysis of NK cell subsets, this activated-PKB also promoted the transition of NKp to iNK and mNK (**Figures 5G–I**). Thus, the ectopic expression of activated-PKB largely corrects the serious defect of PDK1- or/and Rictor-deficient NK cells, suggesting that full activation of PKB by mTORC2 and PDK1 is crucial for NK cell development.

### Phosphoinositide-Dependent Protein Kinase-1 and Mammalian Target of Rapamycin Complex 2 Coordinate to Regulate the Expression of E4BP4 by Activating Protein Kinase B

**Figure 6:**
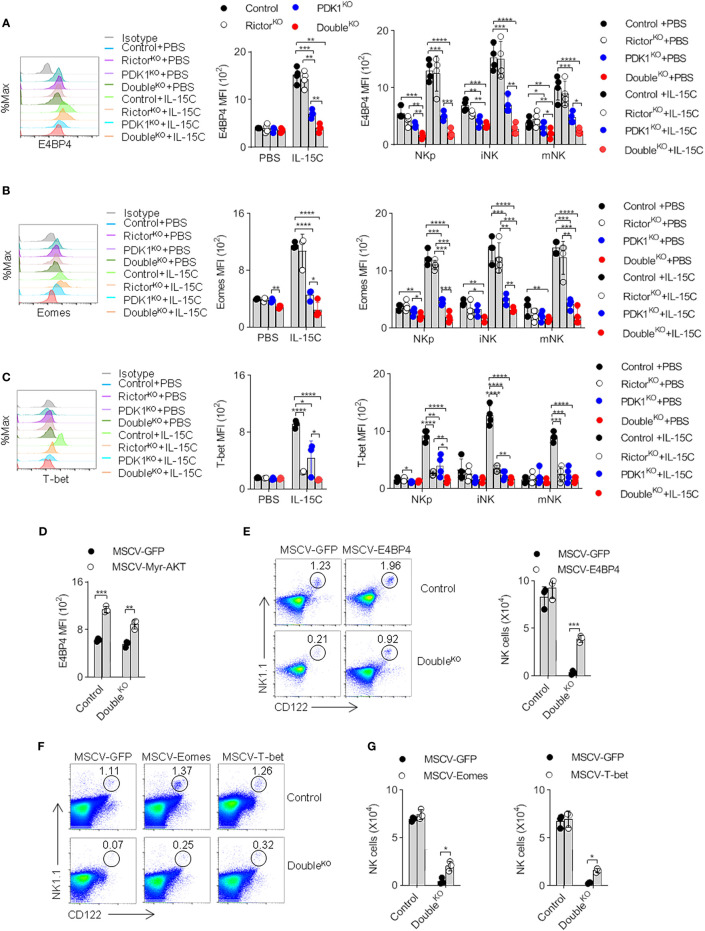
PDK1 and mTORC2 coordinate to regulate the expression of E4BP4 by activating PKB. **(A–C)** The expressions of E4BP4 **(A)**, Eomes **(B)**, and T-bet **(C)** in total NK cells and NK cells at different stages in mice spleen were examined by flow cytometry. Representative overlaid histograms (left), the absolute MFI in total NK cell (middle) and NK cell subsets (left) from mice as annotated. **(D)** The expression level of E4BP4 in NK cell was examined after replenish Myr-AKT to HSC of Double^KO^ mice. **(E–G)** The percentage and number of NK cell were analyzed after replenish E4BP4 **(E)**, Eomes or T-bet **(F, G)** to HSC of Double^KO^ mice. The data represent one of three independent experiments, n≥3 for each experiment, and values are expressed as the mean ± SD. *p < 0.05, **p < 0.01, ***p < 0.001, ****p < 0.0001.

Previous studies reported that PDK1 promotes the expression of major NK cell transcription factor E4BP4 by activating mTOR, which can drive the expression of T-box family transcription factor Eomes (8, 23). Then we investigated whether mTORC2 would further increase the induction of E4BP4. Therefore, the amounts of E4BP4, Eomes and its homolog T-bet in NK cells were determined. Flow cytometry showed that the intracellular amounts of E4BP4 in Control NK cells increased significantly after IL-15C stimulation. Two other transcription factors showed similar increases. The deletion of PDK1 significantly reduced E4BP4, Eomes, and T-bet levels. However, mTORC2 disruption only affected the induction of T-bet. These data reveal different roles of PDK1 and Rictor in transcription factor induction. Nevertheless, we still found that mTORC2 destruction further exacerbated the inability of NK cells with PDK1 deficiency to induce E4BP4 (**Figures 6A–C**). Therefore, mTORC2 may assist PDK1 in regulating the expression of E4BP4.

To verify whether the decreased PKB activity would impair the induction of E4BP4, we reconstructed the active PKB in PDK1/Rictor-deficient HSCs. We found that exogenous supplementation of Myr-PKB mutant could elevate the amount of E4BP4 in NK cells derived from the genetically-modified HSCs and stimulated by IL-15C (**Figure 6D**). Therefore, we believe that PDK1 and mTORC2 coordinate to regulate the expression of E4BP4 by activating PKB.

Next, we investigated whether the ectopic expression of E4BP4 could rescue developmental deficiency caused by the co-deletion of PDK1 and Rictor. First, PDK1/Rictor-deficient HSCs were modified by recombinant E4BP4 retrovirus and then were injected into the recipient mice. Eight weeks later, we found that the frequency and the absolute number of NK cells increased significantly, reaching 70% of the Control level (**Figure 6E**). The replenishment of transcription factor Eomes and T-bet also showed similar results (**Figures 6F, G**). Thus, we think that failure to induce transcription factors, especially E4BP4, may lead to the developmental impairment of NK cells in PDK1/Rictor-deficient mice.

### Phosphoinositide-Dependent Protein Kinase-1 and Mammalian Target of Rapamycin Complex 2 Promote Natural Killer Cell Survival Via Bcl-2

**Figure 7:**
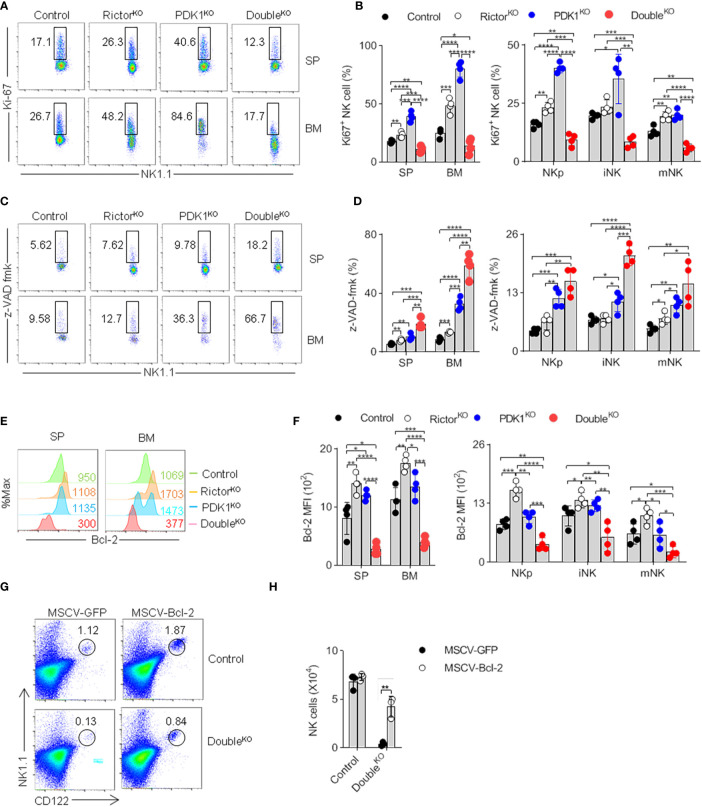
Reduced PKB activation promotes NK cell apoptosis. **(A, B)** Representative flowcytometric plots **(A)** and the percentage **(B)** of total and stage-specific Ki67^+^ NK cells in the SP and BM of indicated mice. **(C, D)** Representative flow cytometric plots **(C)** and the proportion **(D)** of total and stage-specific caspase-positive NK cell in the SP and BM of indicated mice. **(E, F)** Representative overlaid histograms **(E)** and the absolute MFI **(F)** demonstrate the expression levels of Bcl-2 in total and stage-specific NK cells in the spleen and bone marrow of indicated-mice. **(G, H)** Representative flow cytometric results **(G)** and the statistics **(H)** indicate the effect of Bcl-2 on the proportion of NK cell after supplement Bcl-2 to the PDK1/Rictor-deficient mice. The data represent one of three independent experiments, n≥3 for each experiment, and values are expressed as the mean ± SD. *p < 0.05, **p < 0.01, ***p < 0.001, ****p < 0.0001.

PKB controls cell proliferation and survival (24). However, the proportion of Ki67^+^ proliferating NK cells in PDK1- or Rictor-deficient mice appeared to be higher (**Figures 7A, B**). This may be due to the mobilization of NK progenitor as a compensation for severe loss of NK cell at many stages. We thus hypothesized that the decrease of NK cell number might be caused by the increase of apoptosis. Therefore, we assessed whether PDK1- or/and Rictor-deficiency affected caspase activity in NK cells. The results showed that caspase activity increased in NK cells isolated from Rictor-deficient mice, PDK1-deficient mice, and more significantly in PDK1/Rictor-deficient mice, changes in different-stage NK cells are also the same as in total cells (**Figures 7C, D**). These results mean that PDK1 and mTORC2 cooperatively protect NK cells from apoptosis, highlighting an anti-apoptotic role of PKB in NK cells.

Previous studies have shown that overexpression of anti-apoptotic Bcl-2 can overcome NK cell developmental defects (25). We first found that the expression of Bcl-2 in PDK1- and Rictor- double deficient NK cells was much lower than that in Control, PDK1, or Rictor deficient NK cells (**Figures 7E, F**). Therefore, we wondered whether overexpression of Bcl-2 could prevent the increase of NK cell apoptosis in PDK1- and Rictor- double deficient mice. As expected, Bcl-2 retrovirally-delivered in HSCs significantly restored the proportion of NK cells lacking PDK1 and Rictor in recipient mice (**Figures 7G, H**).

In summary, PDK1 and mTORC2 promote NK cell development and survival through PKB, E4BP4, and Bcl-2.

## Discussion

In this study, we demonstrated that the deletion of PDK1 abolished PKB T308 phosphorylation, but enhanced PKB S473 phosphorylation in NK cells. Upon removal of Rictor in PDK1- deficient NK cells, PKB S473 phosphorylation was abolished, which caused further deterioration of NK cell development. In contrast, the ectopic expression of the active form of myristoylated PKB significantly rescued this defect. Mechanistically, PKB regulates NK cell development by promoting transcription factors expression and preventing cell apoptosis.

In the absence of phosphorylation of PKB T308 site in PDK1-deficient mice, the phosphorylation level of S473 site increased to keep PKB activation. The increase of PKB S473 may be caused by the decreased activation of S6 in PDK1- deficient NK cells (**Figure 2E**), which in turn reduces the negative inhibition of S6K on the mTORC2-PKB signal (26), which needs to be further investigated by bypassing the activation of S6 activation. About the increase of p-S6 in Rictor-deficient mice, we think that it may be due to the dual compensatory effect from PDK1. In addition to the phosphorylation of PKB, PDK1 can also activate the PDK1-SGK1 signaling to sustain the PKB-independent mTORC1 activation, which had been tested in tumor cells (27). Of course, this hypothesis needs to be further explored. The increased phosphorylation level of S473 may be the reason why residual NK cells remain survival in PDK1-deficient mice. Because the further deletion of Rictor in PDK1-deficient NK cells could notably diminish the enhanced phosphorylation level of PKB S473, it also accompanied with aggravated NK cell developmental defects, and NK cells were almost undetectable in the double KO mice. The tiny trackable population of NK cells almost were blocked at the NKp stage. Combined with the published data, we suggest that mTORC2 mediated the increased PKB S473 phosphorylation in PDK1-deficient NK cells for compensate of the loss of PDK1.

The full activation of PKB depends on the phosphorylation at T308 and S473 by PDK1 and mTORC2. In this study, we demonstrated that PDK1 and Rictor deficiency caused the decreased phosphorylation of PKB at T308 and S473 in NK cells. The ectopic expression of myristoylated PKB mutant could significantly rescue the defect of NK cell development, suggesting the critical role of PKB as downstream of PDK1 and mTORC2 in NK cells. This needs to be further verified by NK cell-specific deletion of *PKB* genes in the future.

Previous reports showed that PKB could control cell proliferation and survival (24). In this study, we have demonstrated that PDK1 and Rictor play their roles in NK cell survival by keeping the activity of PKB. In both PDK1- and Rictor-deficient NK cells, we could observe significantly increased cell apoptosis. Overexpression of anti-apoptotic Bcl-2 could augment the survival of NK cells in PDK1- and Rictor-deficient mice. This result suggests that PKB promote NK cell survival through anti-apoptotic molecules.

Significant evidence has indicated that transcription factors, including E4BP4, Eomes and T-bet, play a critical role in NK cell development by maintaining the level of CD122 in NK cells. Our previous studies have shown that loss of PDK1 in hematopoietic cells or NKp could inhibit IL-15-induced E4BP4 and Eomes expression in NK cells, thereby decreasing the expression level of CD122 (8, 19). In this study, we confirmed that PDK1 deficiency at the NKp stage inhibited the up-regulation of E4BP4, Eomes and T-bet after IL-15C stimulation. Our findings are in accordance with the previous study showing that Rictor deficiency only decreased the expression of T-bet. In contrast, Eomes expression levels were relatively unaffected in NK cells from Rictor^KO^ mice (10, 11). The level of these transcription factors was lower in the PDK1- and Rictor- double deficiency NK cells after IL-15C stimulation, suggesting a cooperative role of PDK1 and Rictor in IL-15- induced signaling. Ectopic expression of E4BP4, Eomes or T-bet could recover the population NK cells to a significant extent in PDK1- and Rictor-deficient mice, revealing that PDK1 and mTORC2 promote NK cell development through regulating the expression of transcription factors, including E4BP4, Eomes and T-bet. This result also reflects the indispensable role of mTORC2 in the positive feedback loop that involves PDK1-E4BP4-CD122 signal that had been proved critical to NK cell differentiation and development.

In summary, our study revealed the mutual roles of PDK1/mTORC2 signaling during NK cell early development. The early NK cells’ development and survival require fully-activation of PKB, which is maintained by the combined phosphorylation of PDK1 and mTORC2. PDK1/mTORC2 promotes NK cell development and survival through PKB, E4BP4, and Bcl-2.

## Data Availability Statement

The raw data supporting the conclusions of this article will be made available by the authors, without undue reservation.

## Ethics Statement

The animal study was reviewed and approved by Tsinghua University.

## Author Contributions

JH, JZ, YQ, and XH performed the experiments and analyzed the data. JH, JZ, MY, and ZD conceived the hypotheses, designed the experiments, and wrote the manuscript. All authors contributed to the article and approved the submitted version.

## Funding

Research reported in this publication was supported by the Natural Science Foundation of China (to Z.D, 31830027, 81725007, 91942308, and 31821003; to MY, 81771666; to WX, 31700771), National Key Research & Developmental Program of China (2018YFC1003900), Natural Science Foundation of Guangdong Province (to MY, 2019A1515011707), Guangzhou Science and Technology Project (to MY, 201707010395) and the China Postdoctoral Science Foundation (to JH, 2020M670296). The Dong laboratory was also supported by Tsinghua-Peking Center for Life Sciences.

## Conflict of Interest

The authors declare that the research was conducted in the absence of any commercial or financial relationships that could be construed as a potential conflict of interest.

## Supplementary Material

Supplementary Figure 1 Related to **Figure 3**. mTORC2 deficiency aggravates the developmental defect of PDK1-deficient NK cells. **(A)** The gating strategy of NK cell subsets in the SP and BM of annotated mice. NKp (CD3^-^CD122^+^NK1.1^-^CD11b^-^), iNK (CD3^-^CD122^+^NK1.1^+^CD11b^-^), mNK (CD3^-^CD122^+^NK1.1^+^CD11b^+^). **(B, C)** Representative overlaid histograms **(B)** and the absolute MFI **(C)** demonstrate the expression levels of CD122 in NKP cells in the spleen of indicated-mice.
