# Supplementary figures and images for: Full Activation of Kinase Protein Kinase B by Phosphoinositide-Dependent Protein Kinase-1 and Mammalian Target of Rapamycin Complex 2 Is Required for Early Natural Killer Cell Development and Survival
Source: Front Immunol. 2021 Feb 9;11:617404. doi: 10.3389/fimmu.2020.617404 (PMC7901528; doi:10.3389/fimmu.2020.617404)

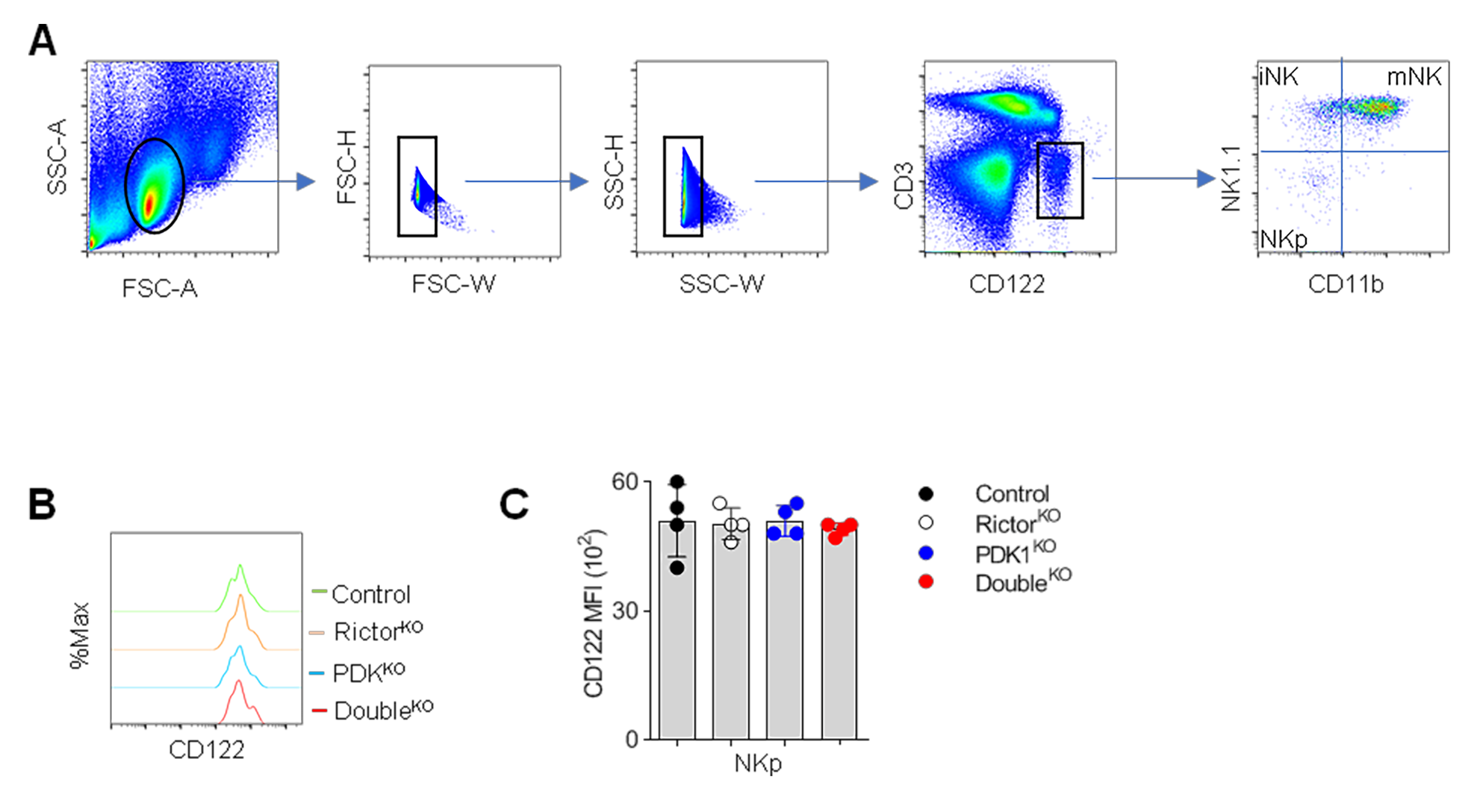

Supplement: Supplementary Figure 1 — Related to Figure 3 . mTORC2 deficiency aggravates the developmental defect of PDK1-deficient NK cells. (A) The gating strategy of NK cell subsets in the SP and BM of annotated mice. NKp (CD3-CD122+NK1.1-CD11b-), iNK (CD3-CD122+NK1.1+CD11b-), mNK (CD3-CD122+NK1.1+CD11b+). (B, C) Representative overlaid histograms (B) and the absolute MFI (C) demonstrate the expression levels of CD122 in NKP cells in the spleen of indicated-mice. [file Image_1.tif]
